# The complete mitochondrial genome of *Pachytriton granulosus* (Chang, 1933) (Amphibia: Caudata: Salamandridae)

**DOI:** 10.1080/23802359.2021.1944368

**Published:** 2021-06-30

**Authors:** Yu Bai, Xuyuan Gao, Jing Chen, Guoyong Li, Jianlin Luo, Hui Wang, Yang Yang, Sheng Liang, Bocheng Ouyang

**Affiliations:** a College of Mathematics & Information Science, Guiyang University, Guiyang, China; b Guangxi Key Laboratory of Biology for Crop Diseases and Insect Pests, Institute of Plant Protection, Guangxi Academy of Agricultural Sciences, Nanning, China; c S&T Park Management Center, Guiyang University, Guiyang, China

**Keywords:** *Pachytriton granulosus*, *Pachytriton labiatus*, *Paramesotriton labiatus*, mitochondrial genome, mitogenome

## Abstract

Complete mitochondrial genome (GenBank accession number MN073499) for *Pachytriton granulosus* (Amphibia: Caudata: Salamandridae) was obtained with Sanger sequencing and assembled manually. The mitogenome consists of a circular DNA molecule of 16,288 bp, with 68.51% AT content. It comprises 13 protein-coding genes, 22 tRNA genes, and 2 rRNA genes. The protein-coding genes have typical ATN (Met) start codons, except *cox1* (GTG as start codon), and are terminated by typical TAN stop codons, except *nad6* (AGA as start codon).

*Pachytriton granulosus* (Chang, 1933) (=*Pingia granulosa*) has become one of the most important species among Chinese salamandrids as a pet (Chang 1933; Nishikawa et al. 2009). According to NCBI Taxonomy database rules, provided taxonomy information was revised. *Pachytriton labiatus* was changed to *Paramesotriton labiatus* (Nishikawa et al. 2011). At present, there are still some papers that use *P. labiatus* (Yotsu-Yamashita et al. 2017; Omura 2019), so for this study, we use *P. granulosus*. Unfortunately, until our study, only a partial mitochondrial genome of *P. granulosus* is available in the GenBank (GenBank accession number: EU880325) (Zhang et al. 2008), length of which is 16,288 bp. The first complete mitochondrial genome (mitogenome) (GenBank accession number: MN073499) for *P. granulosus* was sequenced by us. After our submission to GenBank, Chen et al. from Lishui University submitted the second complete mitogenome (GenBank accession number: MW056200.1) sequenced from Chun’an County, Hangzhou City, China using Illumina sequencing platform, length of which is 16,293 bp.

*Pachytriton granulosus* were first acquired in Lin’an County (106.729562°E, 26.577161°N), Hangzhou City, China, and then bred in our laboratory for several generations. Specimen of adult female *P. granulosus* was obtained in Guiyang University on 8 May 2019, and deposited by Yu Bai (Email: baiyu403@163.com; dk0001@gyu.edu.cn) in the animal specimen room of Guiyang University under the Specimen Accession: GYU-20190508-001. Total Genomic DNA was isolated from the tail tip using the Aidlab Genomic DNA Extraction Kit (Aidlab Co., Beijing, China). 14 pairs of primers designed to match generally conserved regions of target mtDNA published (Zhang et al. 2008) were used to amplify short fragments from 12S, 16S, *cox1*, *cox2*, *nad1*, *nad2*, *nad5*. PCR products were cloned into pMD18-T vector (Takara, JAP) and then sequenced, or sequenced directly by the dideoxynucleotide procedure, using an ABI 3730 automatic sequencer (Applied Biosystems, Foster City, CA, USA). Thirty short sequences were obtained, the length of which ranges from 216 bp to 1000 bp.

The complete mitogenome of *P. granulosus* (MN073499) was assembled manually which consists of a circular DNA molecule of 16,288 bp (33.49% A, 28.38% T, 23.51% C, and 14.62% G; 61.87% AT content). Our mitogenome is as long as the reference mitogenome (EU880325), which is 5 bp shorter than the *P. granulosus* mitogenome of the Chun’an population (MW056200). Using Perna and Kocher’s formula (Perna and Kocher 1995), the AT- and GC-skews of the major strands of the mitogenome were calculated to be approximately 0.083 and −0.233, respectively. The AT-rich region in the mitogenome is 733 bp, with a 78.40% AT content, and is located between the tRNA-Pro and tRNA-Phe.

According to the reference sequence (EU880325) (Zhang et al. 2008), the mitogenome of *P. granulosus* was annotated, which contains 13 protein-coding genes (PCGs), 22 tRNA genes, and 2 rRNA genes. Twelve PCGs (*nad3*, *atp8*, *cox2*, *nad1*, *nad6*, *cob*, *nad4l*, *nad4*, *cox3*, *atp6*, *nad5*, and *nad2*) have typical ATG (Met) start codons, but *cox1* has a GTG start codon. All 13 PCGs have typical TAN stop codons, except *nad6*, which has an AGA stop codon. Seven genes (*nad4l*, *nad5*, *atp8*, *cox1*, *atp6*, *nad2*, and *nad3*) have a TAA stop codon; one gene (*nad1*) has a TAG stop codon; and four genes (*cob*, *cox3*, *nad4*, and *cox2*) have an incomplete stop codon consisting of a T that is completed by the addition of A nucleotides to the 3′ end of the encoded mRNA. The 22 tRNAs are interspersed throughout the coding region, the length of which ranges from 66 bp (tRNA-Cys) to 75 bp (tRNA-Leu). The 16S and 12S are 1560- and 924-bp long, respectively. The maximum overlap length of 15 bp located between *nad5* and *nad6*. The maximum length of the intergenic region is 32 bp, which is located between tRNA-Asn and tRNA-Cys.

To validate the phylogenetic position of *P. granulosus*, mitochondrial PCGs of 12 mitogenomes of Amphibia were used to construct a phylogenetic tree with the MEGA X software (Kumar et al. 2018) via the maximum-likelihood method (Figure 1). Six mitogenomes are from *Pachytriton*, four mitogenomes are from *Paramesotriton*. *Andrias davidianus* mitogenome and *Xenopus laevis* mitogenome were selected as the outgroup. Based on the Bayesian Information Criterions (BIC), General Reversible Mitochondrial (mtREV24) + Gamma distribution (G) (parameter = 0.5178) was chosen as the optimal evolutionary model with 1000 bootstrap replications for the phylogenetic analysis. The phylogenetic tree was divided into two major clades (*Pachytriton* and *Paramesotriton*) and each node of the tree received strong support. *P. granulosus* was clustered into *Pachytriton*. Overall, the new complete mitogenome of *P. granulosus* will contribute to the preservation of its natural resources and evolutionary analysis of this species.

**Figure 1.**
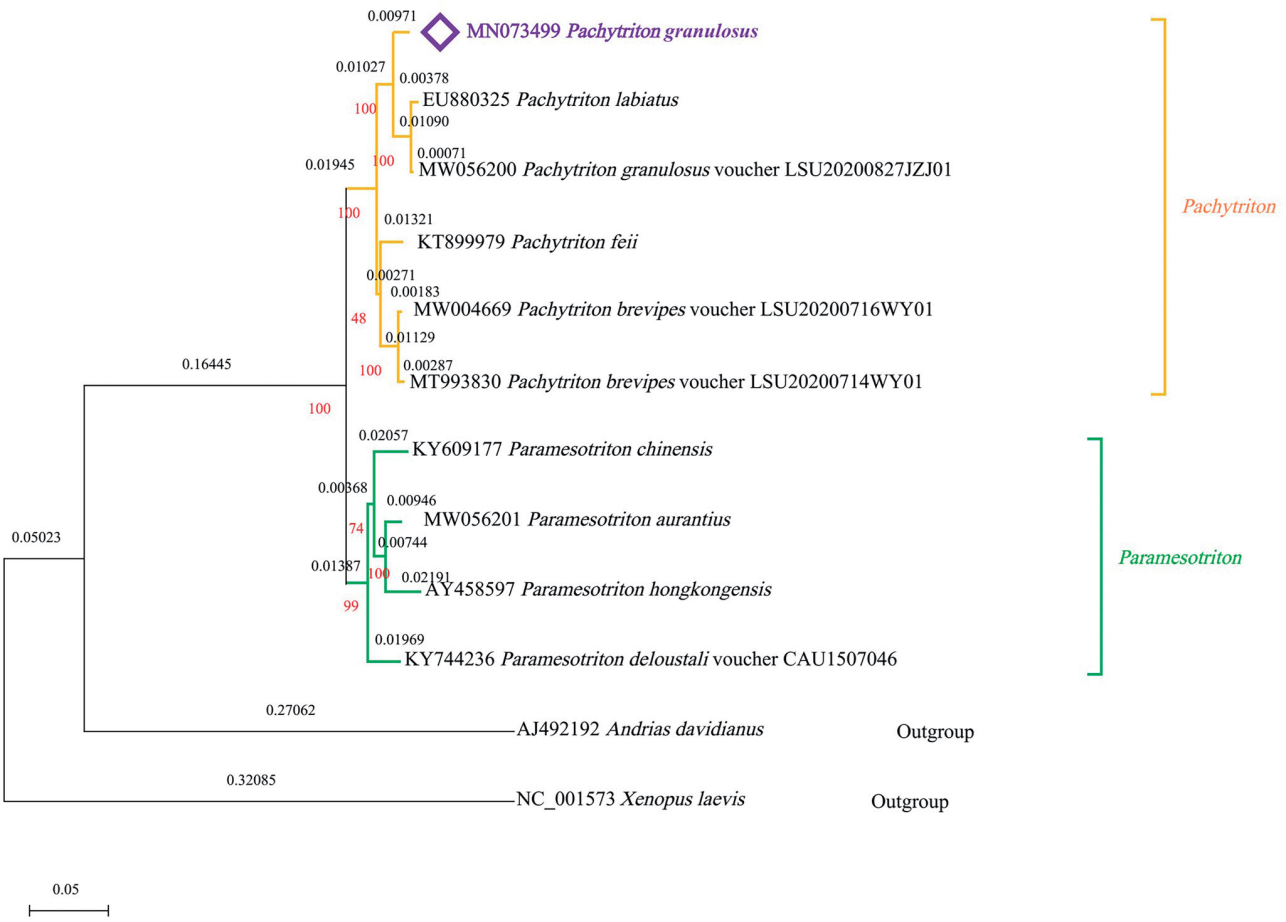
Maximum-likelihood phylogenetic tree of *Pachytriton granulosus* and other species of Amphibia based on the protein-coding regions of their mitogenomes.

The phylogenetic relationship was inferred by using the Maximum Likelihood method and the General Reversible Mitochondrial model. The tree with the highest log likelihood (−22979.39) is shown. The percentage of trees in which the associated taxa clustered together is shown in red numbers below the branches. A discrete Gamma distribution was used to model evolutionary rate differences among sites (five categories (+G, parameter = 0.5178)). The black numbers above the branches indicated Branch lengths which measured in the number of substitutions per site. The percentage of trees in which the associated taxa clustered together is shown below the branches. There was a total of 3805 positions in the final dataset. Evolutionary analyses were conducted in MEGA X. The complete mitogenome of *P. granulosus* from this study was indicated in purple color.

## Data Availability

The genome sequence data that support the findings of this study are openly available in GenBank of NCBI at https://www.ncbi.nlm.nih.gov/nuccore/MN073499.1 under the accession no. MN073499.1.
